# Case Report of RANBP2 Mutation and Familial Acute Necrotizing Encephalopathy

**DOI:** 10.1155/2021/6695119

**Published:** 2021-03-13

**Authors:** Mohamad Paktinat, Kamran Hessami, Soroor Inaloo, Hamid Nemati, Pegah Katibeh, Marzieh Nejabat, Mohammad Hassan Darabi, Ali Hosseini Bereshneh

**Affiliations:** ^1^Neonatology Research Center, Neuroscience Research Center, Shiraz University of Medical Sciences, Shiraz, Iran; ^2^Student Research Committee, Shiraz University of Medical Sciences, Shiraz, Iran; ^3^Maternal-Fetal Medicine Research Center, Shiraz University of Medical Sciences, Shiraz, Iran; ^4^Prenatal Diagnosis and Genetic Research Center, Shiraz University of Medical Sciences, Shiraz, Iran

## Abstract

**Introduction:**

Acute necrotizing encephalopathy (ANE), a rare entity with unique clinical presentation, can be associated significant morbidity and mortality. The majority of ANE reported cases are sporadic. However, reports of extremely rare familial cases are scarce. *Case Presentation*. We described three cases, two siblings and their cousin, affected by ANE, all of them exhibiting RAN-binding protein 2 (RANBP2) gene mutation. They all presented with seizure and decreased level of consciousness. Unlike the siblings, the cousin eventually expired mainly due to the delay in diagnosis, resulting from late presentation of typical brain involvements of ANE in magnetic resonance imaging (MRI).

**Conclusion:**

The presented cases are the first reports of familial ANE in Iran. Attempt was made to raise awareness on this disease, because high clinical suspicion plays an important role in the early diagnosis and proper management of these patients.

## 1. Introduction

Acute necrotizing encephalopathy (ANE) is a rare disorder typically associated with early onset seizures, focal neurological deficits, and rapid deterioration of consciousness, which may ultimately progress to coma [1]. There is an association between ANE and previous upper respiratory infection with organisms like influenza A or parainfluenza, but direct involvement of central nervous system by these agents is unlikely [1].

Findings of neuroradiological studies such as multiple symmetrical lesions involving thalami, brainstem, cerebral white matter, cerebellum, and less frequently spinal cord can assist physicians for the early diagnosis of ANE [2]. The exact pathophysiology of ANE remains to be understood; however, recent studies have suggested that both environmental and genetic factors may play a role in developing ANE [3]. Recently, a number of familial cases of ANE have been identified, all of them showing mutations in RANBP2 gene [4–8].

Considering the rarity of ANE as well as lack of sufficient data on familial cases, we gathered data on three cases of familial ANE in Iran associated with RANBP2 gene mutation.

## 2. Case Presentation

### 2.1. Case 1

**Figure 1:**
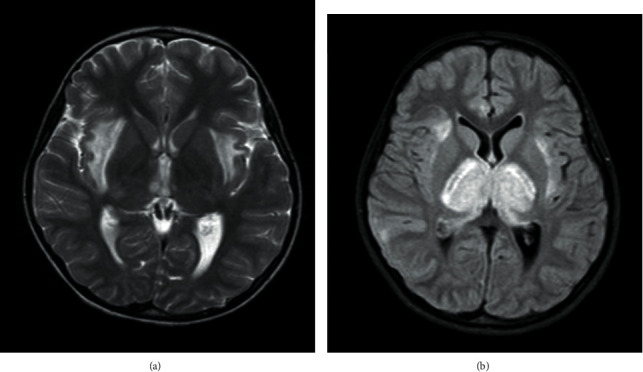
MRI images of case 1 at the (a) first and the (b) second admission.

A 9-year-old girl presented with a decreased level of consciousness, fever, and generalized tonic-colonic seizure with no history of recent traumatic brain injury. Family history was unremarkable. The patient had experienced common cold symptoms which resolved two days prior to admission. Physical examination revealed no sign of focal neurological deficit. Laboratory findings and cerebrospinal fluid (CSF) analysis were within normal ranges. The patient had a similar presentation about 2 years prior to current admission. At the previous presentation 2 years ago, brain magnetic resonance imaging (MRI) revealed hyperintensity of external capsule, cerebellar peduncle, and pons (Figure 1(a)); therefore, she was admitted in pediatric intensive care unit (PICU) for 5 days and received methylprednisolone (2 mg/kg/day) and intravenous immunoglobulin (IVIG) (2 g/kg/day), as a potential case of ANE. Finally, she was discharged with a mild dysarthria considered as neurologic sequelae of ANE. At the current presentation, she was admitted in PICU for 25 days and again received IVIG (2 g/kg/day) and methyl prednisolone (2 mg/kg/day). Brain MRI showed extensive involvement of both basal ganglia, external capsules, both mesial temporal areas, brainstem, and both sides of cerebellar hemisphere (Figure 1(b)). Electroencephalogram (EEG) revealed low-voltage slow wave pattern, suggestive of nonepileptic encephalopathy. Eventually, she was discharged after 1 month with normal cognitive function and her speech ability was relatively recovered with a short period of rehabilitation.

### 2.2. Case 2

**Figure 2:**
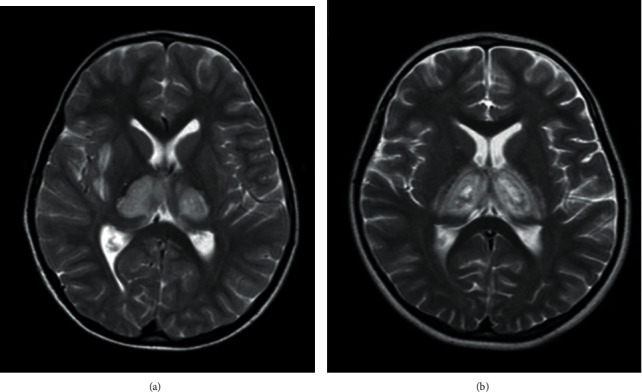
MRI images of case 2 (a) and case 3 (b).

A 4-year-old boy, the brother of the aforementioned case, presented with fever, decreased level of consciousness, and generalized tonic-colonic seizure. Initial examination revealed drowsiness without any focal neurologic deficit. There was no history of recent traumatic brain injury. Family history was positive for ANE in his sister. Laboratory findings were well within normal ranges. CSF analysis did not show pleocytosis, although elevated protein levels were detected. Brain MRI showed increased signal intensity in both thalami, external capsules, midbrain and pons, and also the cortex of the cerebellum (Figure 2(a)). IVIG (2 g/kg/day), methyl prednisolone (2 mg/kg/day), and rituximab (375 mg/m^2^, single dose) were administered, respectively. After a 15-day period of PICU admission, the patient was discharged with no neurologic sequelae. Genetic analysis showed that both siblings (cases 1 and 2) were heterozygous for RANBP2 mutation (c.C1754T: p.T585M). Further genetic investigation revealed the same mutation in their mother, but she had no history of similar symptoms.

### 2.3. Case 3

A 6-year-old boy, the maternal cousin of previous cases, was brought to the emergency room due to generalized tonic-colonic seizure, drowsiness, and fever. Two years earlier, the patient had the similar presentations and was admitted to the pediatric neurology ward with the impression of meningitis and received empirical antibiotic therapy with vancomycin (60 mg/kg/day) and ceftriaxone (200 mg/kg/day). Brain MRI revealed no significant abnormalities, and CSF analysis showed only a slight increase in the protein level. After completing the treatment course, he was discharged with no complication. In the current presentation, however, his symptoms were more severe and he had decreased level of conciseness. Therefore, considering his critical condition and severity of symptoms, the patient was admitted to PICU. Brain MRI revealed bilateral symmetrical relatively expansile, T2-hyperintense lesions and fluid-attenuated inversion recovery (FLAIR) hypersignal change involving both thalami and pons (Figure 2(b)). Unfortunately, the patient was expired two months later, despite receiving immunosuppressive therapies such as methylprednisolone (2 mg/kg/day) and IVIG (2 g/kg/day) and while he was receiving appropriate care in PICU.

## 3. Discussion

For the first time in Iran, we reported three cases of ANE with mutations in the RANBP2 gene. ANE was first described in 1995 [1], since then several nonfamilial (sporadic) case reports of ANE have been published mostly in Eastern Asia.

Neilson and colleagues in 2009 performed a genetic analysis of family members affected with ANE [5]. The authors found a recurrent missense mutation in the RANBP2 gene in family members diagnosed with ANE with an autosomal dominant pattern. Only 40% of the heterozygotes for RANBP2 mutation will manifest an episode of ANE. Thus, the penetrance was thought to be incomplete. This fact may explain the lack of symptoms in the mother of our first two cases.

RANBP2 gene, at a cellular level, is responsible for nucleocytoplasmic trafficking, protein biogenesis, the formation of the mitotic spindle, assembly of the nuclear envelope, and maturation during early mitotic progression [9]. RANBP2 gene mutation is mostly apparent by central nervous system (CNS) involvement. This is attributable to CNS-selective role of RANBP2, which may also underlie the pathogenesis of certain neuropathies [9]. However, the exact pathophysiologic mechanism of RANBP2 mutation and its association ANE remains to be clearly understood.

Similar to previous reports, the patients presented in this study had thalami, external capsules, midbrain, pons, and cerebellum involvements in neuroimaging. However, our third case showed these typical findings only at the second presentation which attributed to the delay in diagnosis.

Treatment of ANE requires critical care management. Methylprednisolone, IVIG, and other immunosuppressants have been used to decrease immune-mediated CNS cell injury in ANE. A previous research suggested that administration of corticosteroids within 24 hours after the onset of symptoms was associated with an improved outcome of children with ANE [10].

Based on previous findings from thirty familial ANE cases, 13% of them expired, 57% had neurological or other types of sequelae, and 30% had complete recovery [7]. Accordingly, poor prognosis of familial ANE highlights the fact that high suspicion and early diagnosis of ANE are critical.

## 4. Conclusion

Familial ANE is a rapidly progressive encephalopathy associated a RANBP2 gene mutation. Physicians must be vigilant to suspect this rare entity especially when it presents with nonspecific neurological symptoms such as the decreased level of consciousness and generalized tonic-colonic seizure. Early identification of typical CNS involvement in neuroradiological study is probably the key factor for the early diagnosis of ANE and improving the prognosis.

## Data Availability

All available information regarding this study has been reported in the manuscript. Any information can be obtained from corresponding author (hessamikamran@gmail.com) based on a reasonable request.
